# Expression and regulation of long noncoding RNAs in TLR4 signaling in mouse macrophages

**DOI:** 10.1186/s12864-015-1270-5

**Published:** 2015-02-05

**Authors:** Ai-Ping Mao, Jun Shen, Zhixiang Zuo

**Affiliations:** Department of Pathology, Committee on Immunology, University of Chicago, Chicago, Illinois the United States; Department of Gastroenterology, Renji Hospital, Shanghai Jiao-Tong University, School of Medicine, Shanghai Institute of Digestive Disease, Shanghai, China; Department of Medicine, University of Chicago, 900 East 57th street, Chicago, IL 60637 USA

**Keywords:** TLR4, LPS, elncRNA, plncRNA, Histone modification

## Abstract

**Background:**

Though long non-coding RNAs (lncRNAs) are emerging as critical regulators of immune responses, whether they are involved in LPS-activated TLR4 signaling pathway and how is their expression regulated in mouse macrophages are still unexplored.

**Results:**

By repurposing expression microarray probes, we identified 994 lncRNAs in bone marrow-derived macrophages (BMDMs) and classified them to enhancer-like lncRNAs (elncRNAs) and promoter-associated lncRNAs (plncRNAs) according to chromatin signatures defined by relative levels of H3K4me1 and H3K4me3. Fifteen elncRNAs and 12 plncRNAs are differentially expressed upon LPS stimulation. The expression change of lncRNAs and their neighboring protein-coding genes are significantly correlated. Also, the regulation of both elncRNAs and plncRNAs expression is associated with H3K4me3 and H3K27Ac. Crucially, many identified LPS-regulated lncRNAs, such as lncRNA-Nfkb2 and lncRNA-Rel, locate near to immune response protein-coding genes. The majority of LPS-regulated lncRNAs had at least one binding site among the transcription factors p65, IRF3, JunB and cJun.

**Conclusions:**

We established an integrative microarray analysis pipeline for profiling lncRNAs. Also, our results suggest that lncRNAs can be important regulators of LPS-induced innate immune response in BMDMs.

**Electronic supplementary material:**

The online version of this article (doi:10.1186/s12864-015-1270-5) contains supplementary material, which is available to authorized users.

## Background

TLR4, a founding member of the TLR family, is a pattern recognition receptor for lipopolysaccharide (LPS) that can induce inflammatory response and cause septic shock [1]. Stimulation of TLR4 by LPS results in the rapid activation of transcription factors, the best characterized of which are interferon regulatory factors (IRFs), the nuclear factor-kappa B (NF-κB) and activator protein 1 (AP-1) families.

In recent years, tens of thousands of long non-coding RNAs (lncRNAs) have been identified in the mammalian genomes, many of which have been implicated in a range of developmental processes and diseases [2-5]. Though most of lncRNAs have been primarily studied in the context of genomic imprinting, developmental process and cancer, lncRNAs are now emerging as important regulators of both innate and adaptive immune responses [6]. Mammalian CD11c + dendritic cells produce many thousands of lncRNAs when stimulated with LPS [7]. The lncRNA Ptprj-as1 is highly expressed in macrophage-enriched tissue and transiently induced by TLR ligands with similar pattern to Ptprj [8]. TLR signaling also induces lncRNA-Cox2, which serve as both repressor and activator of genes through interactions with various regulatory complexes [9]. Li et al. identified a lncRNA *THRIL* regulating TNFα expression through its interaction with hnRNPL during innate activation of THP1 macrophages [10]. Using a global clustering algorithm based on ChIP-seq signals of RNA polymerase II and H3K4me3, Garmire et al. identified a list of putative lincRNAs in mouse macrophages [11]. Most recently, Ilott et al. discovered that both canonical lncRNAs and enhancer lncRNAs regulated the LPS-induced inflammatory response in human monocytes [12]. However, systemic characterization of LPS-regulated lncRNAs in mouse BMDMs is lacking so far.

More and more studies have suggested that although lncRNAs are not specifically targeted in the original array design, a large portion of probes can be reannotated for interrogating lncRNA expression [13-19]. Compared to RNA-seq of low sequencing coverage, microarray data have lower technical variations and higher sensitivity for transcripts with low abundance [20,21], which is a markedly feature of lncRNAs [3]. Additionally, microarray datasets contain strand information, thus allow for interrogating the expression of antisense lncRNAs.

In this study, we aim to explore the activities and potential functions of lncRNAs in LPS-induced innate immune response in mouse BMDMS. To this end, we firstly repurposed different expression microarray platforms to identify lncRNAs from reannotated probes. We then performed an integrative expression analysis of these identified lncRNAs on publicly available expression datasets on LPS-stimulated BMDMS. By using qRT-PCR, we validated the expression changes of some lncRNAs. We classified the lncRNAs to elncRNAs and plncRNAs according to chromatin status defined by relative levels of H3K4me1 and H3K4me3 surrounding transcription start sites. We further examined the correlation of the expression change between lncRNAs and nearest neighboring protein-coding genes. Crucially, several lncRNAs are near to immune response genes, and these pairs are significantly co-expressed, such as lncRNA-Nfkb2/Nfkb2, lncRNA-Rel/Rel. The majority of LPS-regulated lncRNAs have at least one binding site among the transcription factors p65, IRF3, JunB and cJun, further indicating their potential roles in immune response.

## Results

### Reannotating microarray probes for lncRNAs in BMDMs

**Figure 1:**
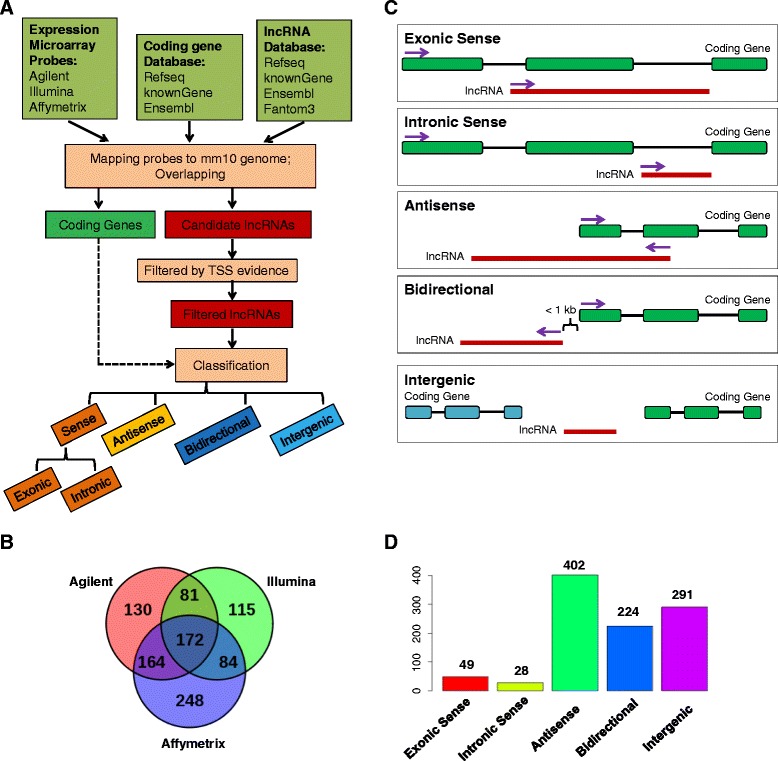
**Mouse microarray probes reannotation and lncRNA classification. (A)** Microarray probes reannotation pipeline for lncRNA. **(B)** Overlap of lncRNAs identified from Agilent, Illumina and Affymetrix platforms. **(C)** Classification of lncRNAs into five classes: exonic sense, intronic sense, antisense, bidirectional and intergenic. **(D)** Bar chart showing the number of lncRNAs in each class for all identified lncRNAs.

To systematically identify LPS-regulated lncRNA profile, we utilized publicly available microarray datasets and reannotated the probes using a comprehensive computational pipeline as illustrated in Figure 1A. From 12 published datasets including six different platforms from Affymetrix, Agilent and Illumina (Additional file 1), we identified 3988 lncRNAs (Additional files 2 and 3). We then incorporated evidence of TSS by TSS-seqs such as CAGE [22] and nanoCAGE [23] or epigenetic markers to filter the lncRNAs. We collected all publicly available mouse TSS-seqs to construct a comprehensive database for mouse gene TSS annotations. Based on the TSS database, we discarded the lncRNAs with no TSS-seq supported or ambiguous TSSs overlapping with neighboring protein-coding genes. Furthermore, we utilized publicly available ChIP-seq data (Additional file 4) to examine the epigenetic markers around the lncRNAs’ TSS region. Those lncRNAs with any epigenetic modifications of H3K4me1, H3K4me3 and PolII were retained. This resulted in 994 reliable lncRNAs with independent transcription evidence (Additional file 5). Although different platforms differed in the lncRNA compositions, they shared a large number of lncRNAs (Figure 1B). We also reannotated the probes to protein-coding genes for all the platforms for further analysis (Additional file 6).

We classified lncRNAs based on their proximity and relative orientation to protein-coding genes (Figure 1C). The 994 lncRNAs with TSS evidence were classified as follows: exonic sense (overlapping a protein-coding gene exons on the same strand), intronic sense (only overlapping a protein-coding gene introns on the same strand), antisense (overlapping a protein-coding gene locus on the opposite strand), biodirectional (on the opposite strand to a protein-coding gene locus and the distance of TSSs is within 1 kb), and intergenic (no-overlapping with a protein-coding gene locus and besides biodirectional) (Figure 1C). The number and distribution of lncRNAs among the different classes were: exonic sense (49, 4.9%), intronic sense (28, 2.8%), antisense (402, 40.4%), bidirectional (224, 22.5%), intergenic (291, 29.3%) (Figure 1D; Additional file 7). Since majority of exonic sense lncRNAs may simply represent fragments of 5′ and 3′ UTRs or nonsense-mediated mRNA decay (NMD) isoforms of protein-coding genes [24], we excluded exonic sense lncRNAs from further analysis.

### LPS-regulated lncRNAs in BMDMs

**Figure 2:**
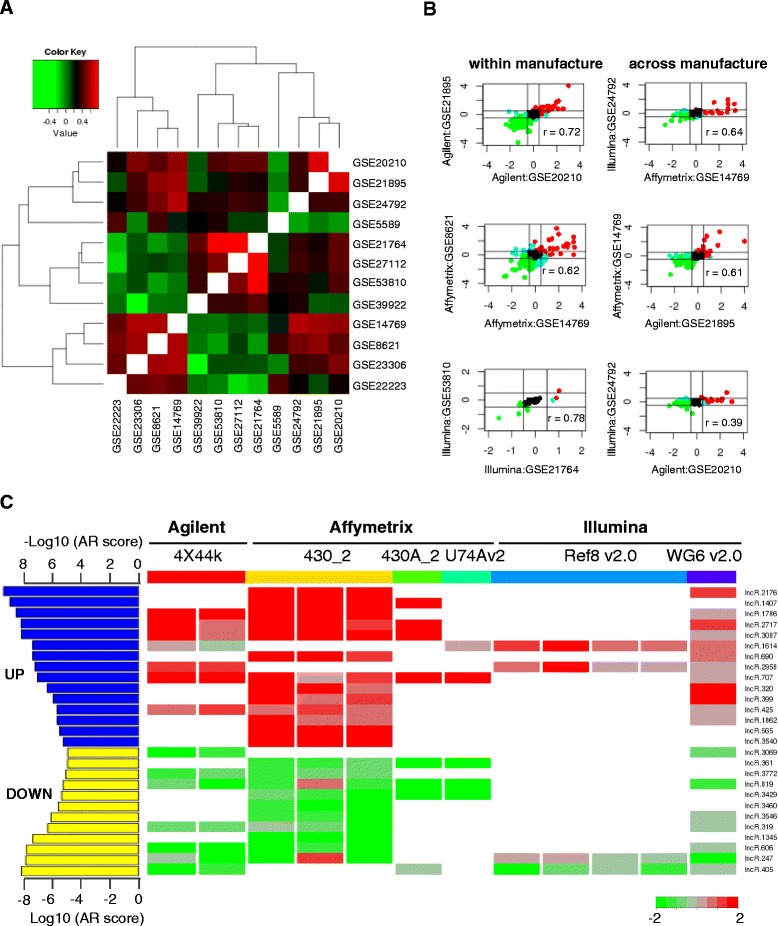
**Correlation between different microarray datasets and integratation of LPS-regulated lncRNAs from all the datasets. (A)** Heatmap showing the Pearson correlation value between all the possible pairs of microarray datasets. **(B)** Represented correlation of the log_2_ expression change of LPS-regulated lncRNAs within (left panel) and across manufactures (right panel). **(C)** The right panel is the heatmap of Log2 expression change of 27 significantly changed lncRNAs upon LPS-stimulation. The fold change was scaled to −2 to 2 by setting all values more than 2 or less than −2 to 2 and −2, respectively. The lncRNAs list was defined by Bonferroni-adjusted p value cutoff 0.05, which was calculated from a robust rank aggregation (RRA) algorithm. On the left panel, the aggregation rank score (AR score) from RRA was shown. AR score indicated the integrated rank from the integrated analysis of fold change from 12 different microarray datasets. For upregulated lncRNAs, −Log10(AR score) was used while for downregulated lncRNAs, Log10(AR score) was used.

**Figure 3:**
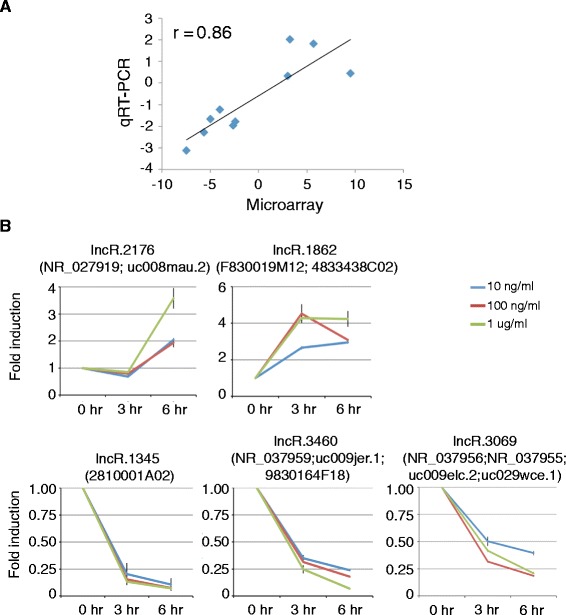
**qRT-PCR validations of LPS-regulated lncRNAs from integrated microarray datasets. (A)** Correlation analysis of averaged log_2_ (fold change) derived from qRT-PCR and +/− log_10_(AR score) derived from integrated microarrays as shown in Figure 2C. **(B)** The relative expression levels of two upregulated and three downgregulated lncRNAs identified by integrated analysis in BMDMs stimulated with 10 ng/ml, 100 ng/ml and 1 ug/ml LPS for 0, 3 and 6 hours.

Based on the reannotated probes, we obtained LPS-regulated lncRNAs in BMDMs from individual datasets, the majority of which have LPS stimulation time points ranging from 3 to 6 hours. We then used Pearson correlation analyses to evaluate the consistency of microarrays within each manufacture and across manufactures. The correlation of all pairs of microarrays is in the range from −0.26 to 0.80 (Figure 2A). With only a few exceptions, majority of the pairs are showing significant positive correlation. The pairs that had low correlation only because there were limited overlaps between them. The expression pattern of lncRNAs in different datasets from the same manufacture had remarkably high correlation (Figure 2A and B). Moreover, the expression of overlapped lncRNAs could also be largely validated by cross-manufacture datasets (Figure 2A and B). The consistency of lncRNA expression represented by multiple probes from different platforms suggests the reliability of probe reannotation. Though different platforms of LPS-regulated lncRNA expression have overall agreements, some varieties also exist. To integrate these microarrays in an unbiased manner, we exploited a recently published robust rank aggregation algorithm [25] (Additional file 8). Different platforms for protein-coding genes had even stronger consistency (Additional file 9), and we also integrated their expression using the same strategy (Additional file 10). To validate the results, we randomly selected 10 LPS-regulated lncRNAs to do quantitative RT-PCR (qRT-PCR) in BMDMs stimulated by different concentrations of LPS for 0, 3 and 6 hours (Additional file 11). The fold changes determined by qRT-PCR were strongly correlated with integrated microarray analysis result (Figure 3A, r = 0.86). Using bonferroni-adjusted P value 0.05 as the cutoff, we identified 15 upregulated and 12 downregulated lncRNAs upon LPS stimulation in BMDMs (Figure 2B; Additional file 12). Markedly, the expression change of two upregulated and three downregulated lncRNAs significantly stimulated by LPS was confirmed by qRT-PCR (Figure 3B). As expected, the fold changes of lncRNAs showed some dependence on LPS stimulation time and concentration. Taken together, we accurately recapitulated the lncRNA expression changes upon LPS stimulation.

### Chromatin signatures separate elncRNAs and plncRNAs

**Figure 4:**
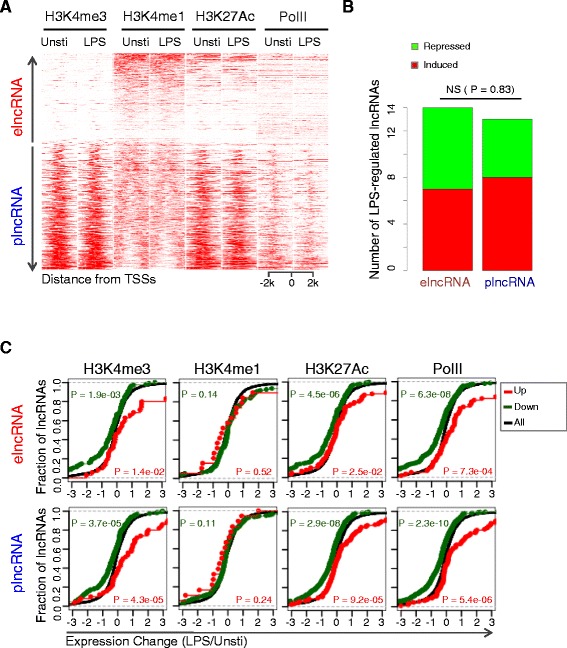
**Chromatin signatures surrounding TSSs separate lncRNAs into elncRNAs and plncRNAs. (A)** Heatmap presenting the read intensities of H3K4me3, H3K4me1, H3K27Ac and PolII modification across a 4 Kb interval centered on TSSs of the 994 lncRNAs before and after LPS stimulation in BMDMs (the y-axis indicates 994 lncRNAs and the label was not shown, refer to Additional file 13). **(B)** The numbers of LPS-upregulated and -downregulated lncRNAs among elncRNAs and plncRNAs showed by bar chart. The P value was calculated by chi-square test. **(C)** Empirical cumulative distribution function (ecdf) plot was showing to indicate the correlation between the change epigenetic markers (H3K4me3, H3K4me1, H3K27Ac and PolII) and the expression change of their corresponding lncRNAs. Upper panel is elncRNAs, while lower panel is plncRNAs. The red and blue curves represent lncRNAs that were marked with increased and decreased epigenetic markers, respectively. Black curve represent all lncRNAs. Based on the knowledge that all these four epigenetics marks are active markers that positively correlated with expression changes, one-sided KS-test was performed to evaluate the difference between red curve and black curve, and similarly the difference between blue curve and black curve was evaluated.

Previous studies have suggested that the ratio of H3K4me1/H3K4me3 around TSSs can separate lncRNAs into elncRNAs and plncRNAs [26-30]. To classify the lncRNAs identified from microarrays accordingly, we utilized publicly available histone modification ChIP-seq data (Additional file 4). We calculated the relative ratio of H3K4me1/H3K4me3 in a four Kb window centered on TSSs. Of note, 370 (37.2%) of 994 lncRNAs showed enhancer-like features (H3K4me1/H3K4me3 high), while the remaining lncRNAs displayed promoter histone signatures (H3K4me1/H3K4me3 low) (Figure 4A; Additional file 13). LPS stimulation had marginal effect on the relative ratio of H3K4me1/H3K4me3, thus lncRNAs displayed similar enhancer-like or promoter-associated histone signatures in both unstimulated and LPS stimulated BMDMs (Figure 4A). As expected, both elncRNAs and plncRNAs were enriched with H3K27Ac modification, a well-accepted mark of biological activity (Figure 4A). These LPS-regulated plncRNAs and elncRNAs can be either induced or repressed by LPS stimulation, and no difference was detected between plncRNAs and elncRNAs (Figure 4B, p = 0.83, chi-square test).

It has been well established that histone modification changes are associated with changes of lncRNA expression, which is confirmed in our finding. We observed that differences in H3K4me3 and H3K27Ac were positively correlated with changes in both elncRNAs and plncRNAs expression (Figure 4C). H3K4me1 was not associated with expression change upon LPS stimulation for both elncRNAs and plncRNAs (Figure 4C). PolII signal for LPS regulated elncRNAs and plncRNAs was significantly changed upon LPS stimulation (Figure 4C).

### Correlation between lncRNA and neighboring protein-coding gene expression

**Figure 5:**
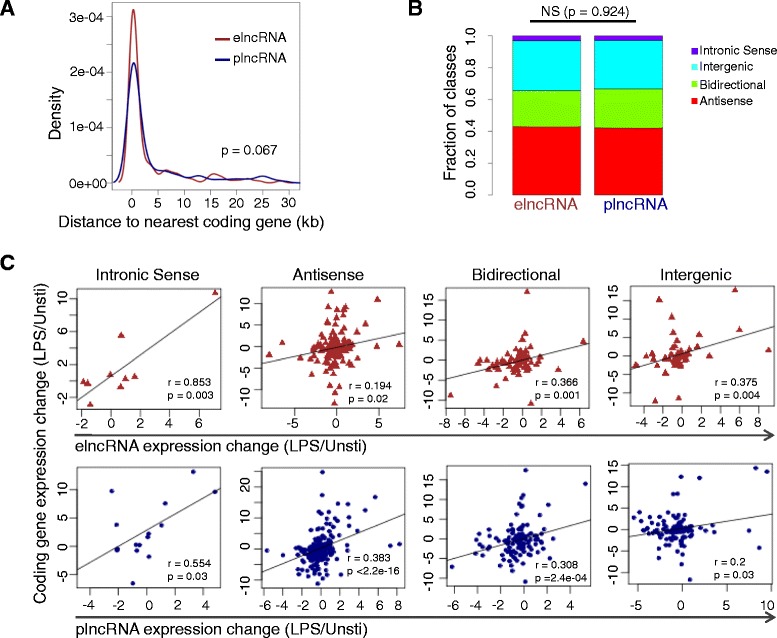
**Comparison of distance and correlation to closest neighboring protein-coding gene between elncRNAs and plncRNAs. (A)** Density distribution of distances from the nearest neighboring protein-coding genes for elncRNAs and plncRNAs (P value is from t-test). **(B)** Percentages of sense, antisense, bidirectional and intergenic lncRNAs for elncRNAs and plncRNAs. **(C)** Expression change correlation of closest neighboring protein-coding gene and elncRNAs (upper panel) and plncRNAs (lower panel).

LncRNAs have been reported to coordinate the regulation of neighboring protein-coding genes through a locus control process [31]. We assessed their relative distance and correlation of expression changes to neighboring gene for elncRNAs and plncRNAs, respectively. Interestingly, when considering intergenic and bidirectional lncRNAs only, although not significant, elncRNAs were in a closer proximity to protein-coding genes than plncRNAs (Figure 5A, p = 0.066, t test). The elncRNAs and plncRNAs had similar compositions of lncRNA classes (Figure 5B, p value = 0.93, chi-square test). Although the distance to nearest protein-coding genes for plncRNAs is not as close as elncRNAs, all the intronic sense, antisense, bidirectional and intergenic elncRNAs/plncRNAs are significantly co-expressed with neighboring protein-coding genes (Figure 5C, all p < 0.05, KS test). Intronic sense lncRNA expression change has the strongest correlation with protein-coding gene neighbors, compared to other three classes (Figure 5C), suggesting that intronic sense lncRNAs are probably often co-transcribed with closest mRNA genes.

### LPS-regulated lncRNAs closely related to inflammatory response

**Figure 6:**
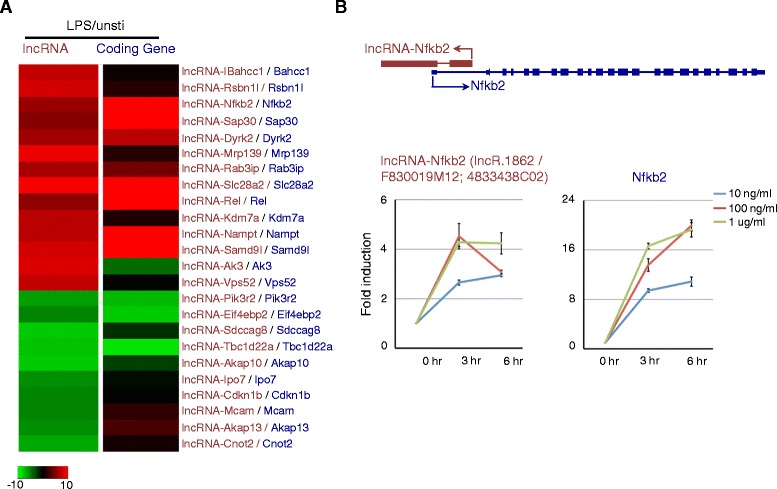
**Co-regulation between lncRNA and neighboring protein-coding gene expression upon LPS stimulation in BMDMs. (A)** Heatmap showing the integrated expression change of LPS-regulated lncRNAs and nearest protein-coding gene neighbors. **(B)** qRT-PCR validation of three lncRNA-protein-coding gene pairs co-regulated upon LPS stimulation in BMDMS.

We found 24 out of 27 LPS-regulated lncRNAs were adjacent to or overlapped with protein-coding genes. Adapting the previously proposed nomenclature [32], we renamed the 24 lncRNA genes based on their neighboring protein-coding genes (Additional file 14). Notably, the majority of these lncRNAs (19/24) were positively correlated with neighboring protein-coding genes, while only 5 of 24 were negatively correlated (Figure 6A). Of particular interest was LPS-regulated lncRNAs paired with neighboring protein-coding genes known to play roles in inflammatory response, such as NFKB pathway genes Nfkb2 and Rel (Figure 6A). Using qRT-PCR, we validated the co-expressed lncRNA-Nfkb2 and NFkb2 pairs in BMDMs stimulated with various concentrations of LPS for 0, 3 and 6 hours (Figure 6B).

### LPS-induced transcriptional regulation of lncRNAs in BMDMs

**Figure 7:**
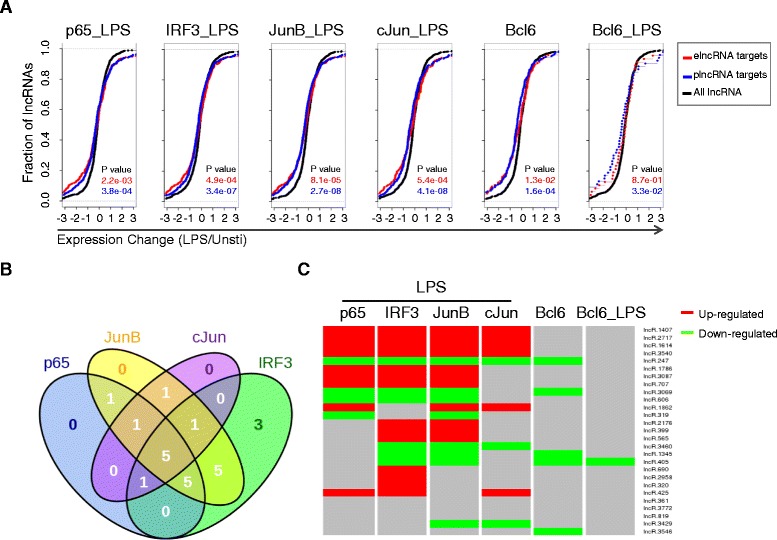
**The cooperated regulation of transcription factors on lncRNAs upon LPS stimulation in BMDMs. (A)** Ecdf plots demonstrate the expression difference between transcription factor targets (red: elncRNA; blue: plncRNA) and all lncRNAs (black) (P values are from two-sided KS-test). **(B)** Venn diagrams of the numbers of overlapped lncRNAs bound by p65, IRF3, JunB and cJun upon LPS stimulation in BMDMs. **(C)** Heatmap showing that whether LPS-upregulated (red) and LPS-downregulated (green) lncRNAs were bound by transcription factors p65, IRF3, JunB, cJun and Bcl6. The red and green colors mean the lncRNAs were bound by the corresponding transcription factor, while the gray color means not bound.

In resting BMDMs, the transcription factors p65, IRF3 and AP-1 family members JunB and cJun are sequestered in cytoplasm. Upon LPS stimulation, these transcription factors are rapidly translocated to nucleus, where they act alone or together with one another to bind numerous gene loci to regulate gene expression. To determine whether these transcription factors were required for transcriptional regulation of lncRNAs, we took advantage of published ChIP-seq data and reanalyzed the peaks. Firstly, we found that both LPS-regulated elncRNAs and plncRNAs were enriched for binding sites of the four transcription factors. Interestingly, the transcription factors binding sites were enriched in lncRNAs whose expression was either increased or decreased after LPS stimulation, for both elncRNAs and plncRNAs (Figure 7A). Bcl6 is a transcriptional factor that binds genes and broadly constrains the inflammatory response through cistromic antagonism of a TLR-NF-κB network [33]. We detected the similar enrichment pattern for Bcl6 before and after LPS stimulation.

**Figure 8:**
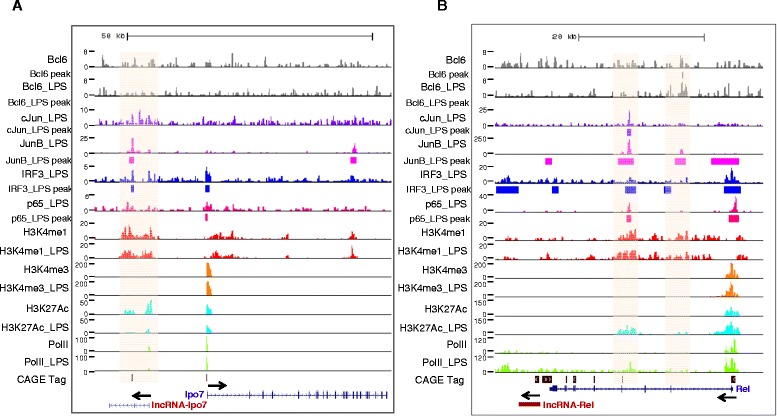
**The transcription factors binding and histone modifications. (A)** lncRNA-Ipo7 and Ipo7. **(B)** lncRNA-Rel and Rel. Arrows indicate the direction of transcription.

Of the 27 LPS-regulated elncRNAs and plncRNAs identified in BMDMs, 23 were bound by at least one of the four transcription factors (p65, IRF3, JunB and cJun) (Figure 7B). p65, IRF3, JunB and cJun bound to 13, 20, 19 and 9 lncRNAs in BMDMs exposed to LPS, respectively (Figure 7B). Of note, 5 lncRNAs were bound by all the four transcription factors. Different transcription factor binding sites of lncRNAs were within average 2 Kb, suggesting that these proteins acted together to regulated lncRNA expression. Interestingly, the majority of Bcl6 binding sites (80% (4/5) and 100% (1/1) in LPS-unstimulated and -stimulated BMDMs, respectively) colocalized with at least one of IRF3, p65, JunB and cJun sites after LPS stimulation (Figure 7C). Above findings indicated that IRF3, p65 and AP-1 family member JunB and cJun were the major transcription factors that acted in a synergetic manner and regulated lncRNAs expression in TLR4 signaling pathway, while Bcl6 antagonized some lncRNA binding sites to prevent hyperimmune response. For example, lncRNA-Ipo7 was co-bound by IRF3 and JunB within a small window (Figure 8A). Out of two potential promoter regions for lncRNA-rel, one was bound by p65, IRF3, JunB and cJun in BMDMs after LPS stimulation, and the other one was bound by IRF3 and JunB after LPS stimulation, while the binding site was antagonized by Bcl6 under rest condition (Figure 8B).

## Discussion

Previous studies mainly focused on the study of LPS-regulated protein-coding genes but ignored the function of lncRNAs involved. To explore the potential role of lncRNAs in the activation of TLR4 signaling, we constructed a comprehensive bioinformatics pipeline to reannoate probes to lncRNA from literature expression microarray datasets in BMDMs. Giving that large number of such datasets are available in public repositories, the pipelines we generated will be useful for reannotating array probes to address different biological questions.

Our integrated lncRNA and protein-coding gene expression profiles are valuable resources for understanding the LPS-stimulated program, as well as their co-regulation. Having established that LPS induced widespread changes in the expression of lncRNAs in mouse macrophages situated close to differentially expressed immune response-related genes, it was important to determine whether these were functionally relevant. Of great interest was the identification of differentially expressed lncRNAs that are located close to two members of Ref/Nfkb family, Nfkb2 and Ref, which are classical proinflammatory transcription factors known to play critical roles in both innate and adaptive immune response. Nfkb2 was reported to be upregulated upon LPS stimulation in human monocytes [34]. Our qRT-PCR experiments confirmed the co-expression of lncRNA-Nfkb2 and Nkfb2. It is unlikely that the co-regulation of lncRNAs and Ref/Nfkb family was a random phenomenon since two members of this family were found to be co-regulated with lncRNAs. Given the importance of Ref/Nfkb family in immune response, a further examination of the function and mechanism for their co-located and co-expressed lncRNAs is worth doing. However, our study has limitations to detect known LPS-regulated lncRNAs due to the lack of probes. Previous study indicated that Cox2 and lncRNA-Cox2 were markedly induced after TLR4 stimulation in BMDMs [9]. Due to the lack of probes for lncRNA-Cox2, we did not detect lncRNA-Cox2 in this study. We performed qRT-PCR to confirm the co-expression of Cox2 and lncRNA-Cox2 (Additional file 15). It should be noted that we applied a stringent strategy to derive a confident list of LPS-regulated lncRNAs. Some interesting lncRNAs are also filtered out, such as lncRNA-Lyn-intron1. Previous study has demonstrated that lncRNA-Lyn spans the first exon and first intron region of Lyn and the expression is increased along with Lyn upon LPS stimulation in BMDMs [9]. We identify a new Lyn associated lncRNA, lncRNA-Lyn-Intron1 (lncR.2430; Additional file 3), located at the first intron of Lyn and 25 Kb away from lncRNA-Lyn, is also upregulated (Additional file 15). This lncRNA was filtered out because of no clear TSS evidence.

Recent investigation in erythroid cells has suggested that the lncRNAs transcripts are almost evenly divided between elncRNAs and plncRNAs differentiated by chromatin signatures of H3K4me3 and H3K4me1 surrounding transcription start sites [27]. Consistent with this observation, we found that BMDMs expressed elncRNAs and plncRNAs were also evenly distributed. A number of elncRNAs and plncRNAs can be regulated by LPS stimulation. Nevertheless, plncRNAs are more inclined to downregulation upon LPS stimulation compared to elncRNAs. Several previous studies suggested that lncRNA expression changes are regulated by epigenetic mechanism including histone modifications such that H3K27ac, H3K4me3 and H3K36me3 are related to enhancer activity [15]. Similarly, we demonstrated that histone modifications also play important roles in the regulation of lncRNAs upon LPS stimulation in BMDMs. We found that H3K4me3 and H3K27Ac are associated with directionally consistent changes in not only elncRNAs, but also plncRNAs expression. Our studies demonstrate that although distance to nearest neighboring is much nearer in elncRNAs compared to plncRNAs, these both of two kinds of lncRNAs significantly co-expressed with neighboring protein-coding genes. Bidirectional transcription has been shown to be a defining feature of a subset of active enhancers in mouse cortical neurons and human fetal lung fibroblasts [35,36]. We have shown that the transcription of bidirectional plncRNAs, as well as elncRNAs, were LPS-stimulation dependent in mouse BMDMs.

The gene program stimulated in TLR4 signaling pathway requires the coordinative activation of transcription factors, of which the most well characterized are p65, IRF3, and AP-1 family members JunB and cJun. Here we demonstrate for the first time that these transcription factors also bind to lncRNAs and regulate their expression upon LPS stimulation. Also, the regulation does not differ between elncRNAs and plncRNAs. The majority of LPS-regulated lncRNAs are bound with at least one of these transcription factors. Markedly, we also identify the up-regulated and down-regulated lncRNAs that are bound by all the four transcription factors, suggesting the widely cooperation of these transcription factors. Recent study suggests that Bcl6 antagonizes p65 bindings under rest condition to prevent the hyper-activation of inflammatory genes [33]. Interestingly, we found that Bcl6 also binds to a portion of lncRNAs and the binding sites can overlap with not only p65, but also IRF3, JunB and cJun. We speculate these transcription factors may regulate lncRNAs in a similar manner to protein-coding genes upon LPS stimulation in BMDMs.

## Conclusions

Taken together, we have provided a valuable resource of LPS-regulated lncRNA expression profile, together with many potential co-regulated candidate protein-coding genes. Among them, we have identified lncRNAs such as lncRNA-Nfkb2 and lncRNA-Rel that are upregulated along with their corresponding protein-coding genes, which are crucial genes in immune response. Although the mechanisms are currently unknown, we speculate that many of the identified elncRNAs and plncRNAs are important participants of LPS-stimulated innate immune response. We also established an integrative microarray analysis pipeline, which opens new avenues for repurposing published genomic data to study the functions and mechanisms of lncRNAs in interested biology fields.

## Methods

### Re-annotation of array probes

The mouse gene annotations were collected from four sources: NCBI RefSeq [37], UCSC knownGene [38], FANTOM3 [39] and Ensembl [40]. For NCBI RefSeq, the mm10 version of mouse refGene was downloaded, and transcripts beginning with “NR” were treated as non-coding RNAs, while transcripts beginning with “NM” were treated as coding RNAs. For UCSC knownGene, the mm10 version was downloaded and transcripts annotated with “noncoding” were considered as non-coding RNAs, while transcripts annotated with “coding” were considered as coding RNAs. A stringent set of FANTOM3 non-coding RNAs was selected based on the conservation and noncoding votes. The fasta sequences of the stringent FANTOM3 non-coding RNAs were aligned against mm10 genome using blat [41] to obtain mm10 annotation of FANTOM3 non-coding RNAs. For Ensembl, the release 77 for mouse was downloaded, and the transcripts annotated with “protein_coding” were treated as coding RNAs, otherwise as non-coding RNAs. We excluded non-coding RNAs with length < 200 nt from the four sources, and defined others as long non-coding RNAs (lncRNAs). We reannotated probes of six different platforms from Affymetrix, Agilent and Illumina arrays (Additional file 1: Table S1) for lncRNAs using the following procedure. Firstly, the bed format annotations of all array probes were generated. The mm10 bed files for Affymetrix arrays were directly downloaded from the Affymetrix website (http://www.affymetrix.com). For Agilent and Illumina arrays, we obtained the probe sequences from the Agilent website (http://www.agilent.com) and NCBI GEO database (http://www.ncbi.nlm.nih.gov/gds), respectively. The probe sequences were mapped against mm10 genome using blat, and the bed format annotations of the best hits were generated. Secondly, the bed format annotations of probes were intersected with lncRNA annotations and coding gene annotations to obtain lncRNA probes and coding gene probes, respectively. BedTools [42] were utilized to achieve this end. To avoid hybridizations, the probes that were mapped to multiple lncRNA annotations or coding gene annotations were removed. The summary information of probe reannotation result for each array platform is shown in Additional file 2. As a result, 3988 unique lncRNAs were obtained. The detailed reannotations of all probes of the six platforms for lncRNAs are shown in Additional file 3. The detailed reannotations of all probes of the six platforms for coding genes are shown in Additional file 6.

### Determination of transcriptional start sites (TSSs)

We used CAGE [22] and nanoCAGE [23] TSS-seq to determine genome-wide TSSs for mouse genome as described elsewhere [27]. To obtain full annotation of TSSs for mouse genome, we collected all the available TSS-seq from DBTSS [43] and NCBI SRA [44]. The mm9 bed files of TSS-Seq sequences were downloaded from DBTSS (ftp://ftp.hgc.jp/pub/hgc/db/dbtss/dbtss_ver8), and then were converted from mm9 to mm10 using the UCSC liftOver tools (http://genome.ucsc.edu/cgi-bin/hgLiftOver). The fastq files of mouse TSS-Seq sequences (GSE49459 and GSE39849) were downloaded from NCBI SRA using SRA toolkit. Then the TSS-seq sequences were mapped to mm10 genome using bwa [45]. A perl script was written to integrate all the TSS-Seq to obtain the TSS regions. Briefly, the 5′ end position of each TSS-Seq read was extracted as TSS. TSSs closer than 20 bp and derived from the same strand were clustered. Clusters within 400 bp of each other and on the same strand were further grouped as a TSS region. The TSS regions with less than 20 tags supported were discarded, thus 160116 TSS regions were retained.

### Filter lncRNAs by TSS evidence

We associated the lncRNAs reannotated from arrays to the 160116 TSS regions using BEDTools [42]. The lncRNA region plus 30 Kb upstream/downstream regions were used to scan for the TSS regions. As a result, 25100 TSS regions were found to locate nearby the 3575 lncRNAs that have determined TSSs. ChIP-seq raw reads for H3K4me3, H3K4me1 and H3K27Ac histone modifications and RNA PolII in unstimulated/LPS BMDMs were downloaded from NCBI GEO database (http://www.ncbi.nlm.nih.gov/gds/) (Additional file 4). The raw reads were aligned to mm10 mouse genome using bowtie 1.0.1 [46] with the –m reporting option set to 2. The peaks of histone modifications and PolII were called using MACS [47] following published parameters [48]. To further refine the TSS regions for lncRNAs, we integrated chromatin signatures such as histone modifications and PolII occupancy nearby lncRNAs to obtain a list of active and reliable TSS regions. The candidate TSS regions for lncRNAs were examined for the peaks of H3K4me3, H3K4me1 and PolII. Only those TSS regions with at least one peak of them were retained, which resulted in 7474 TSS regions associated with 2629 lncRNAs. To further refine the TSS regions for the lncRNAs, we excluded the ambiguous TSS regions which may be overlapped with the neighboring coding gene TSS regions, resulting in 1503 TSS regions for 994 lncRNAs, as listed in Additional file 5.

### lncRNA classification

The lncRNA classification method was adopted from a previous study [32]. The lncRNAs were classified according to their relation with neighbor coding genes. The neighbor coding genes of lncRNAs were selected on the basis of either the nearest distance to the lncRNA or the longest overlapping regions. The lncRNAs with distance to their neighbor coding gens shorter than 1 kb and with different orientation as their neighbor coding genes were categorized as bidirectional. The lncRNAs that have not any overlap with the neighbor coding genes and not belong to bidirectional were categorized as intergenic. The lncRNAs overlapping with their neighbor coding genes were categorized as genic. The genic lncRNAs where were further classified as sense or antisense according to the orientation relation with neighbor coding genes. The classification of all lncRNAs in this study is shown in Additional file 7.

### Expression analysis of LPS stimulated BMDM

We collected expression array datasets from the studies on the investigation of transcriptional profile in mouse BMDMs, which resulted in 12 arrays from six array platforms (Additional file 1). The expression datasets were downloaded from NCBI GEO repository [49]. For the Affymetrix and Illumina array datasets, we downloaded the probe-level preprocessed expression matrix file directly. For the Agilent array datasets, we downloaded the raw data, and used the R package “Agi4x44Preprocess” [50] to preprocess the raw data. KNN method [51] was used to fill the missing values for each preprocessed expression matrix. Then the probe-level expression matrix was transformed to gene/lncRNA level using the reannotated information as shown in Additional files 3, 4, 5 and 6. The average expression was taken if multiple probes were mapped to the same transcript. For each transcript, the average expression for unstimulated group and LPS stimulated group were calculated separately. Then the log2 fold change between the two groups for each transcript was calculated. The expression profiles in the 12 studies for all the lncRNAs are shown in Additional file 8. The expression profiles in the 12 studies for all the coding genes are shown in Additional file 10. We employed a recently published robust rank aggregation algorithm [25] to integrate these 12 expression profiles in an unbiased manner. A P value was obtained for each transcript to represent the upregulation and downregulation under LPS stimulation, respectively. The bonferroni-adjusted p value cutoff 0.05 was used to select the significantly changed lncRNAs/coding genes.

### Preparation of BMDMs

Bone marrow cells were harvested from the femurs and tibias of 8-week-old C57BL/6 mice. BMDMs were generated by culture of bone marrow cells in RPMI medium containing 10% of FBS and10 ng/ml of recombinant M-CSF (R&D Systems, cat. no. 416-ML-010) for 7 days. Differentiated BMDMs were then stimulated with different concentrations of LPS (Sigma, cat. no. L3024) for 0, 3 and 6 hours in RPMI medium. All the mice were raised in a specific pathogen–free environment at the University of Chicago, and experiments were performed in accordance with the guidelines of the Institutional Animal Care and Use Committee.

### RNA isolation and qRT-PCR

Total RNA from different time points of LPS stimulated BMDM was prepared with TRIZOL (Invitrogen, cat. no. 15596026), according to the manufacture’s instruction. The cDNA was synthesized from total RNA using SuperScript First-Strand Synthesis System (Invitrogen, cat. no. 11904018). Q-PCR was performed using SYBR Advantage Premix (Clontech, cat. no. 639676) in Strategene Mx3500 thermocycler. The corresponding primers were listed in Additional file 16.

### Determining chromatin signatures at lncRNA TSSs

We did a quantity assessment for the enrichment of H3K4me3, H3K4me1, H3K27Ac and PolII around each lncRNA TSS region using in-house R script utilizing Rsamtools [52] in R. The relative enrichment of H3K4me3 and H3K4me1 surrounding the transcription start sites of the lncRNAs (−2 to 2 Kb) was calculated to define elncRNA and plncRNA as previously described [27]. Heatmaps of the elncRNA and plncRNA histone modification profiles were generated using heatmap.2 function in R package “gplots” [53].

### Association of lncRNA loci with transcription factor binding sites

ChIP-seq raw reads for transcription factors p65, IRF3, JunB, cJun and Bcl6 were downloaded from NCBI GEO database (http://www.ncbi.nlm.nih.gov/gds/) (Additional file 11). The raw reads were aligned to the mm10 mouse genome build using bowtie 1.0.1 [46] with the –m reporting option set to 2. The bedgraphs of ChIP-seq were generated using HOMER, where the total number of aligned reads was normalized to 10 million. The peaks of transcription factors were called using MACS [47] following published parameters [48]. The transcription factor binding sites were associated to lncRNA promoter-proximal region (−10 kb to 10 kb from TSS) using BEDtools [42].

### Statistical analysis

Pearson product–moment correlation coefficient was used to measure the linear correlation. Student’s t test was used to evaluate the significance of difference for distance to neighboring gene between elncRNAs and plncRNAs. Kolmogorov-Smirnov (K-S) test was performed to evaluate the significance of difference between ecdf curves. All the statistical analyses were performed in R using the built-in packages.

## Additional files

Additional file 1: Table S1. The NCBI GEO dataset accession numbers for selected microarrays are shown. Additional file 2: Table S2. The number of microarray probes reannotated for the lncRNAs from Agilent, Illumina and Affymetrix platforms. Additional file 3: Table S3. The detailed information of lncRNAs identified from Agilent, Illumina and Affymetrix platforms. Additional file 4: Table S4. The NCBI GEO dataset accession numbers for selected ChIP-seq data are shown. Additional file 5: Table S5. The list of filtered lncRNAs identified from Agilent, Illumina and Affymetrix platforms. Additional file 6: Table S6. Reannotation of microarray probes for the protein-coding genes. Additional file 7: Table S7. The classification of lncRNAs in BMDMs to exonic sense, intronic sense, antisense, bidirectional and intergenic. Additional file 8: Table S8. The expression profile of lncRNAs in BMDMs integrated from the microarray datasets. Additional file 9: Figure S1. Correlation between different microarray datasets and integratation of LPS-regulated protein-coding genes from all the datasets. (A) Correlation of the log_2_ expression change of LPS-regulated protein-coding genes within (upper panel) and across platforms (lower panel). (B) Heatmap of the expression profile of integrated LPS-regulated protein-coding genes across 12 datasets in BMDMs. Additional file 10: Table S9. The expression profile of protein-coding genes in BMDMs integrated from the microarray datasets. Additional file 11: Table S10. qRT-PCR validation of an random selection of LPS-regulated lncRNAs. Additional file 12: Table S11. Significantly changed lncRNAs upon LPS-regulation. P value was derived from robust rank algorithm. Bonferroni method was used to adjust p value. Adjusted p value 0.05 was used as the cutoff to derive the significantly changed lncRNAs. TSS evidence was also shown. Additional file 13: Table S12. Annotating lncRNAs by chromatin signatures, neighboring protein-coding genes and transcription factors binding sites. Additional file 14: Table S13. The integrated expression change of LPS-regulated lncRNAs and their closest neighboring genes. LPS-regulated lncRNAs are renamed according to the nearest neighboring protein-coding genes. Additional file 15: Figure S2. The co-regulation of lncRNA and local mRNA gene pairs stimulated by 10 ng/ml, 100 ng/ml and 1 ug/ml LPS for 0, 3 and 6 hours in BMDMs. Additional file 16: Table S14. The list of qPCR primers used.
