# Supplementary material for: Expression and regulation of long noncoding RNAs in TLR4 signaling in mouse macrophages
Source: BMC Genomics. 2015 Feb 5;16(1):45. doi: 10.1186/s12864-015-1270-5 (PMC4320810; doi:10.1186/s12864-015-1270-5)
Supplement: Additional file 1: Table S1. — The NCBI GEO dataset accession numbers for selected microarrays are shown. [file 12864_2015_1270_MOESM1_ESM.docx]

| Platform | Accession NO. | LPS time | LPS concentration | Mouse strain | Reference |
| --- | --- | --- | --- | --- | --- |
| Agilent-014868 4x44K G4122F | GSE21895 | 4 hr | 10 ng/ml | C57BL/6 |  |
| Agilent-014868 4x44K G4122F | GSE20210 | 6 hr | 100 ng/ml | C57BL/6 | (Ghigo *et al*, 2010) |
| Affymetrix 430 2.0 | GSE23306 | 4 hr | 100 ng/ml | C57BL/6 | (Satoh *et al*, 2010) |
| Affymetrix 430 2.0 | GSE14769 | 4 hr | 10 ng/ml | C57BL/6 | (Litvak *et al*, 2009) |
| Affymetrix 430 2.0 | GSE8621 | 3 hr | 100 ng/ml | C57BL/6 | (Mages *et al*, 2007) |
| Affymetrix 430A 2.0 | GSE22223 | 5 hr | 1 ug/ml | C57BL/6 | (Rao *et al*, 2010) |
| Affymetrix U74A v2 | GSE5589 | 3 hr | 100 ng/ml | 129x  C57BL/6 | (Kasmi *et al*, 2006) |
| Illumina Ref-8 v2.0 | GSE39922 | 8 hr | 100 ng/ml | C57BL/6 | (Wang *et al*, 2012) |
| Illumina Ref-8 v2.0 | GSE27112 | 3 hr | 100 ng/ml | C57BL/6 | (Cheng *et al*, 2011) |
| Illumina Ref-8 v2.0 | GSE21764 | 4 hr | 100 ng/ml | C57BL/6 | (Nicodeme *et al*, 2010) |
| Illumina Ref-8 v2.0 | GSE53810 | 4 hr | 10 ng/ml | C57BL/6 | (Sung *et al*, 2014) |
| Illumina WG-6 v2.0 | GSE24792 | 6 hr | 100 ng/ml | C57BL/6 | (Barish *et al*, 2010) |

Table S1. NCBI GEO dataset accession numbers for selected microarrays are shown.
