# Supplementary material for: Expression and regulation of long noncoding RNAs in TLR4 signaling in mouse macrophages
Source: BMC Genomics. 2015 Feb 5;16(1):45. doi: 10.1186/s12864-015-1270-5 (PMC4320810; doi:10.1186/s12864-015-1270-5)
Supplement: Additional file 2: Table S2. — The number of microarray probes reannotated for the lncRNAs from Agilent, Illumina and Affymetrix platforms. [file 12864_2015_1270_MOESM2_ESM.docx]

|  | **Agilent 4x44k** | **Affymetrix Mouse430_2** | **Affymetrix Mouse430A_2** | **Affymetrix MG_U74Av2** | **Illumina Ref-8 v2.0** | **Illumina WG-6 v2.0** |
| --- | --- | --- | --- | --- | --- | --- |
| **Probes mapped to mm10 genome** | 41166 | 44097 | 22338 | 12060 | 25694 | 41519 |
| **Probes mapped to lncRNA (combined)** | 4440 | 6429 | 3209 | 1581 | 1538 | 4066 |
| NCBI RefGene | 1704 | 2284 | 976 | 479 | 819 | 1685 |
| Fantom3 | 1261 | 2782 | 1410 | 573 | 506 | 1142 |
| UCSC knownGene | 1065 | 1620 | 426 | 211 | 133 | 877 |
| Ensembl | 2180 | 2700 | 1178 | 673 | 327 | 1839 |
| **Probes mapped to coding gene (combined)** | 31006 | 33236 | 21171 | 11267 | 25097 | 35455 |
| NCBI RefGene | 27468 | 31090 | 20843 | 11025 | 24578 | 31018 |
| UCSC knownGene | 29095 | 31852 | 20975 | 11100 | 24798 | 33649 |
| Ensembl | 30026 | 32543 | 21025 | 11150 | 24757 | 34112 |
| **Probes mapped to lncRNA (no overlap with Coding Gene)** | 2431 | 2086 | 330 | 260 | 238 | 1899 |
| **lncRNAs** | 2141 | 1773 | 262 | 218 | 220 | 1689 |
| **Combined unique lncRNAs** | 3988 | | | | | |
| **lncRNAs filtered by TSS/epigenetic evidence** | 994 | | | | | |

**Table S2 Summary of probes reannotation**
