# Supplementary material for: Expression and regulation of long noncoding RNAs in TLR4 signaling in mouse macrophages
Source: BMC Genomics. 2015 Feb 5;16(1):45. doi: 10.1186/s12864-015-1270-5 (PMC4320810; doi:10.1186/s12864-015-1270-5)
Supplement: Additional file 4: Table S4. — The NCBI GEO dataset accession numbers for selected ChIP-seq data are shown. [file 12864_2015_1270_MOESM4_ESM.docx]

| Accession NO. | LPS time | LPS concentration | Antibody | Mouse strain | Reference |
| --- | --- | --- | --- | --- | --- |
| GSM419049 | - | - | Bcl6 | C57BL/6 | (Barish *et al*, 2010) |
| GSM851557 | - | - | Bcl6 |  |  |
| GSM611114 | 3 hr | 100 ng/ml | Bcl6 |  |  |
| GSM611114 | 3 hr | 100 ng/ml | Bcl6 |  |  |
| GSM611116 | 3 hr | 100 ng/ml | p65 |  |  |
| GSM611117 | 3 hr | 100 ng/ml | p65 |  |  |
| GSM940935 | - | - | PolII | C57BL/6 | (Ostuni *et al*, 2013) |
| GSM940936 | 4 hr | 10 ng/ml | PolII |  |  |
| GSM940913 | - | - | H3K4me3 |  |  |
| GSM940914 | - | - | H3K4me3 |  |  |
| GSM940915 | 4 hr | 10 ng/ml | H3K4me3 |  |  |
| GSM940902 | - | - | H3K27Ac |  |  |
| GSM940903 | - | - | H3K27Ac |  |  |
| GSM940904 | 4 hr | 10 ng/ml | H3K27Ac |  |  |
| GSM940891 | - | - | H3K4me1 |  |  |
| GSM940892 | - | - | H3K4me1 |  |  |
| GSM940893 | 4 hr | 10 ng/ml | H3K4me1 |  |  |
| GSM1022319 | 4 hr | 10 ng/ml | JunB |  |  |
| GSM788653 | 3 hr | 100 ng/ml | cJun | C57BL/6 | (Uhlenhaut *et al*, 2013) |
| GSM925279 | 3 hr | 100 ng/ml | IRF3 |  |  |
