# Supplementary material for: Expression and regulation of long noncoding RNAs in TLR4 signaling in mouse macrophages
Source: BMC Genomics. 2015 Feb 5;16(1):45. doi: 10.1186/s12864-015-1270-5 (PMC4320810; doi:10.1186/s12864-015-1270-5)
Supplement: Additional file 9: Figure S1. — Correlation between different microarray datasets and integratation of LPS-regulated protein-coding genes from all the datasets. (A) Correlation of the log2 expression change of LPS-regulated protein-coding genes within (upper panel) and across platforms (lower panel). (B) Heatmap of the expression profile of integrated LPS-regulated protein-coding genes across 12 datasets in BMDMs. [file 12864_2015_1270_MOESM9_ESM.pptx]

## Slide 1
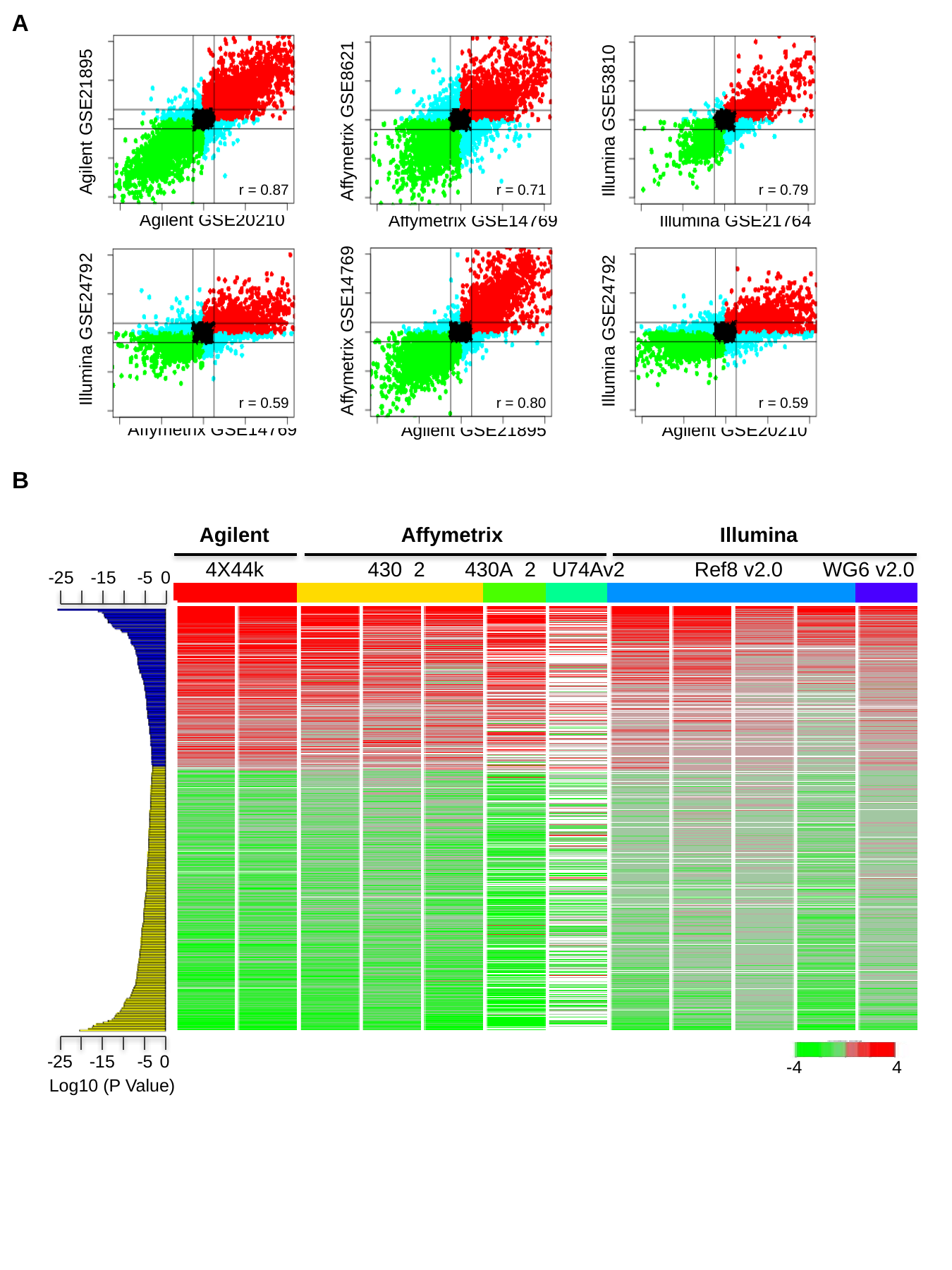

A
Affymetrix GSE8621
Illumina GSE53810
Agilent GSE21895
Affymetrix GSE14769
Illumina GSE21764
Agilent GSE20210
Affymetrix GSE14769
Illumina GSE24792
Illumina GSE24792
Affymetrix GSE14769
Agilent GSE21895
Agilent GSE20210
r = 0.87
r = 0.71
r = 0.79
r = 0.59
r = 0.80
r = 0.59
B
Agilent
Affymetrix
Illumina
4X44k
430_2
430A_2
U74Av2
Ref8 v2.0
WG6 v2.0
-25
-15
-5
0
-25
-15
-5
0
Log10 (P Value)
-4
4
