# Supplementary material for: Expression and regulation of long noncoding RNAs in TLR4 signaling in mouse macrophages
Source: BMC Genomics. 2015 Feb 5;16(1):45. doi: 10.1186/s12864-015-1270-5 (PMC4320810; doi:10.1186/s12864-015-1270-5)
Supplement: Additional file 15: Figure S2. — The co-regulation of lncRNA and local mRNA gene pairs stimulated by 10 ng/ml, 100 ng/ml and 1 ug/ml LPS for 0, 3 and 6 hours in BMDMs. [file 12864_2015_1270_MOESM15_ESM.pptx]

## Slide 1
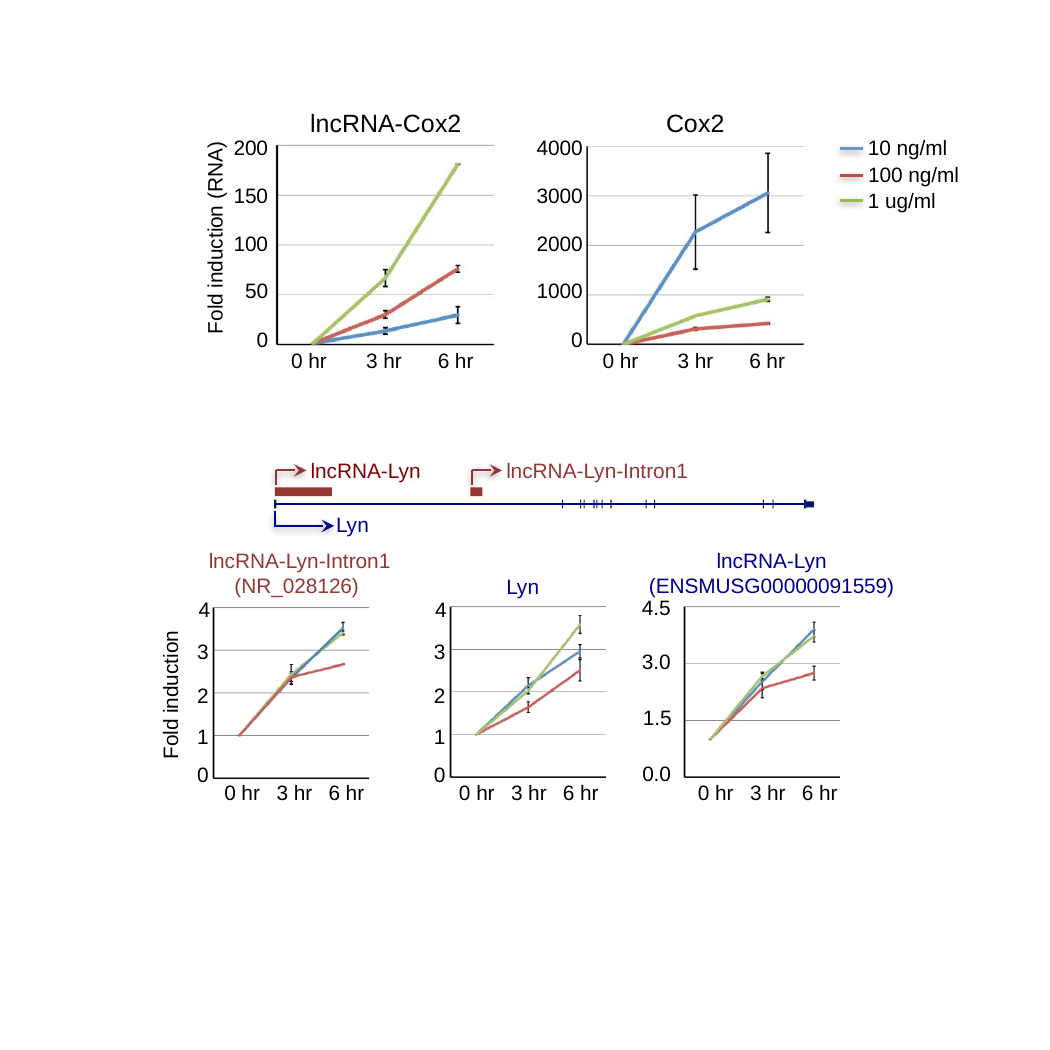

Cox2
4000
3000
2000
1000
 0
0 hr
3 hr
6 hr
lncRNA-Cox2
 200
 150
 100
 50
 0
0 hr
3 hr
6 hr
10 ng/ml
100 ng/ml
1 ug/ml
Fold induction (RNA)
lncRNA-Lyn
lncRNA-Lyn-Intron1
Lyn
lncRNA-Lyn
(ENSMUSG00000091559)
4.5
3.0
 1.5
0.0
3 hr
0 hr
6 hr
lncRNA-Lyn-Intron1
 (NR_028126)
4
 3
 2
 1
 0
3 hr
0 hr
6 hr
Lyn
4
 3
 2
 1
 0
3 hr
0 hr
6 hr
Fold induction
